# Glutaredoxin 2 Reduces Asthma-Like Acute Airway Inflammation in Mice

**DOI:** 10.3389/fimmu.2020.561724

**Published:** 2020-11-03

**Authors:** Eva-Maria Hanschmann, Carsten Berndt, Christina Hecker, Holger Garn, W. Bertrams, Christopher H. Lillig, Christoph Hudemann

**Affiliations:** ^1^ Department of Neurology, Medical Faculty, Heinrich-Heine University Düsseldorf, Düsseldorf, Germany; ^2^ Biochemical Pharmacological Center (BPC)–Translational Inflammation Research Division, Philips Universität Marburg, Member of the German Center for Lung Research (DZL) and the Universities of Giessen and Marburg Lung Center (UGMLC), Marburg, Germany; ^3^ Institute for Lung Research, Universities of Giessen and Marburg Lung Center, Philipps-University Marburg, Member of the German Center for Lung Research (DZL), Marburg, Germany; ^4^ Institute for Medical Biochemistry and Molecular Biology, University of Greifswald, Greifswald, Germany; ^5^ Department of Dermatology and Allergology, Philipps Universität Marburg, Marburg, Germany; ^6^ Institute of Laboratory Medicine and Pathobiochemistry, Molecular Diagnostics, Philipps University Marburg, Marburg, Germany

**Keywords:** airway inflammation, asthma, redox, oxidative dysbalance, reactive oxygen species

## Abstract

Endogenous redox systems not only counteract oxidative damage induced by high levels of hydroxyl radicals (OH·) under pathological conditions, but also shape redox signaling as a key player in the regulation of physiological processes. Second messengers like hydrogen peroxide and nitric oxide, as well as redox enzymes of the Thioredoxin (Trx) family, including Trxs, glutaredoxins (Grxs), and peroxiredoxins (Prxs) modulate reversible, oxidative modifications of proteins. Thereby redox regulation is part of various cellular processes such as the immune response and Trx proteins have been linked in different disorders including inflammatory diseases. Here, we have analyzed the protein distribution of representative oxidoreductases of the Trx fold protein family—Trx1, Grx1, Grx2, and Prx2—in a murine model of allergic asthma bronchiale, as well as their potential therapeutic impact on type-2 driven airway inflammation. Ovalbumin (OVA) sensitization and challenge using the type-2 prone Balb/c mouse strain resulted in increased levels of all investigated proteins in distinct cellular patterns. While concomitant treatment with Grx1 and Prx2 did not show any therapeutic impact on the outcome of the disease, Grx2 or Trx1 treatment before and during the OVA challenge phase displayed pronounced protective effects on the manifestation of allergic airway inflammation. Eosinophil numbers and the type-2 cytokine IL-5 were significantly reduced while lung function parameters profoundly improved. The number of macrophages in the bronchoalveolar lavage (BAL) did not change significantly, however, the release of nitric oxide that was linked to airway inflammation was successfully prevented by enzymatically active Grx2 *ex vivo*. The Grx2 Cys-X-X-Ser mutant that facilitates de-/glutathionylation, but does not catalyze dithiol/disulfide exchange lost the ability to protect from airway hyper reactivity and to decrease NO release by macrophages, however, it reduced the number of infiltrating immune cells and IL-5 release. Altogether, this study demonstrates that specific redox proteins and particular enzyme activities protect against inflammatory damage. During OVA-induced allergic airway inflammation, administration of Grx2 exerts beneficial and thus potentially therapeutic effects.

## Introduction

The term asthma circumscribes several clinical phenotypes that are all characterized by chronic airway inflammation. Variable expiratory airflow limitations are caused by structural rearrangements such as mucus hypersecretion and infiltrating inflammatory cells (e.g. eosinophils and lymphocytes). During the last two decades, modulation of cell activation or suppression by reactive oxygen or nitrogen species has been shown to play a vital role in the regulation of innate and adaptive immune processes. Specific second messengers such as hydrogen peroxide (H_2_O_2_) and nitric oxide (NO) participate in cellular redox regulation that is mediated by enzymes of the Thioredoxin (Trx) family (1). The development and progression of asthma is characterized by increased levels of specific reactive oxygen and nitrogen species (e.g. OH^·^ and ONOO^-^), leading to the oxidation and nitration of various proteins with high specificity (2–4). Local levels of the tripeptide glutathione (GSH) were also shown to be altered in asthma. GSH is vital for enzyme-based redox signaling, cell polarization, and antioxidant defense due to its ability to provide redox-equivalents during its switch from reduced GSH to the oxidized glutathione disulfide (GSSG). In healthy adults, GSH concentrations are up to 100-fold higher in the epithelial lining fluid, the oxygen-delivering border between host and environment compared to the overall plasma concentrations. In asthmatics, a global baseline perturbation with regards to thiol availability and thiol redox signaling can be detected. Downstream redox systems including the Trx and GSH/Glutaredoxin (Grx) systems can subsequently be affected in allergic patients (4).

The mammalian Trx protein family comprises more than 50 members that are ubiquitously expressed and display individual subcellular distribution patterns and catalyze different types of reactions. Here, we have focused on four mainly cytosolic proteins that were also shown to translocate to the nucleus or extracellular space upon distinct stimuli (5). Trx, Grx1, Grx2, and Prx2 were shown to play a role in cell proliferation, migration, and inflammation, for instance *via* regulation of the transcription factor NFkB or TLR4 signaling. They catalyze different types of reactions.

In fact, the functional and mechanistic spectrum of Trx family proteins is based on their Cys-X-X-Cys active site motif and comprises e.g. protein disulfide exchange reactions, (de-)glutathionylation, (de-)nitrosylation, and sulfenylation (6). Grxs can catalyze GSH-dependent reduction of disulfides and glutathione-mixed disulfides functioning as an electron donor, e.g., for ribonucleotide or sulfate reduction, and controlling the levels of protein GSH-mixed disulfides. Disulfide exchange reactions depend on the dithiol mechanism that requires both active site Cys residues, whereas the (de-)glutathionylation depends on the monothiol mechanism and thus only requires the N-terminal Cys residue (7). In contrast to Grx1 very little is known about Grx2 in allergic inflammation. Peroxiredoxins (Prxs) catalyze the reduction of peroxides and function in redox signaling by oxidation of target proteins (8). In many pulmonary diseases such as COPD (chronic obstructive pulmonary disease), asthma, acute lung injury, cystic and idiopathic pulmonary fibrosis, and lung cancer as well as the ovalbumin (OVA) based asthma mouse model elevated expression of Trx family proteins was found in affected tissues [see rev. (9–12)]. Knock-out mice of Prx2 exhibit induced inflammatory vasculopathy, ER stress and autophagy (13).

Due to the involvement of different specialized cell types, specific reactive molecules, redox signaling, and protein inactivation, asthma is a very complex pathology. We need new therapeutic strategies to counteract the chronic inflammation and tissue remodeling. Given the importance of redox enzymes during allergic airway diseases, the goal of this study was to investigate the distribution of Grx1, Grx2, Trx1, and Prx2 in the OVA-based model of acute allergic airway inflammation in Balb/c mice and to analyze the therapeutic potential of administration of recombinant proteins.

## Material and Methods

### General Methods

Chemicals were purchased from Sigma (St. Louis, USA), unless otherwise stated, and were of analytical grade or better. The generation of the antibodies (Grx1, Grx2, Trx1, Prx2) has been described before in the redox atlas of the mouse. Antibodies were produced in rabbits and were thoroughly validated in mouse tissues, including lung. Specificity validation was done by immunoblotting and antigen-blocking with recombinant proteins (14). Female BALB/c mice aged 6–8 weeks were obtained from Harlan Winkelmann (Borchen, Germany) and housed four animals per cage in a 12/12 h light/dark cycle with food and water available *ad libitum*. All experimental procedures were approved by the local animal ethics committee (#48/2011) and met German and international guidelines.

### Murine Ovalbumin (OVA)-Induced Acute Asthma Model

5- to 7-week old female Balb/c mice were sensitized to OVA by intraperitoneal (i.p.) injection of 10 μg OVA (grade VI; Sigma) without adjuvant in a total volume of 200 μl PBS on days 0, 7, and 14. This “Sensitization” phase was followed by 20 min 1% OVA or PBS aerosol exposure on days 24, 25, 26 (“Challenge”). I.p. protein injections (40 µg/shot) of Grx1, Grx2, Trx1, or Prx2 were performed in the lower right quadrant of the abdomen at days 23 to 27 once daily (see **Supplementary Figure 1**, n = 8–16 per group). Forty-eight hours after the last aerosol challenge, mice were deeply anesthetized by intraperitoneal injection (i.p.) of 300 mg/kg ketamine hydrochloride (Ketamin, Inresa Arzneimittel GmbH, Freiburg, Germany) plus 30 mg/kg Xylazine (Rompun, Bayer Health Care, Leverkusen, Germany) prior to exsanguination by cutting the abdominal aorta.

### Protein Expression and Purification

The plasmids for the recombinant expression of human Trx1, Grx1, Grx2, and Prx2 in *E. coli* were described before (15–17). All proteins were expressed as His-Tag proteins in *E. coli* and were purified by immobilized metal affinity chromatography and FPLC as described previously (18) (see **Supplementary Figure 2**). Note that this expression system gives rise to redox-active enzymes, as shown for Trxs, Grxs, and Prxs before (18). For the cell culture experiments mouse Grx2 was also expressed as His-Tag protein in ClearColi® BL21(DE3) electrocompetent cells (Lucigen), BL21(DE3) derived cells with several key mutations that result in a lack of outer membrane agonists for hTLR4/MD-2 activation and therefore contain significantly reduced endotoxicity (19). Proteins were purified by immobilized metal affinity chromatography (GE Healthcare), endotoxins were removed using high capacity endotoxin removal columns according to manufacturer’s instructions (Thermo Scientific, USA) and were tested for their endotoxin levels using the HEK-BlueTM LPS Detection Kit2 (InvivoGen, San Diego, USA).

### Bronchoalveolar Lavage (BAL) Acquisition and Cytology

By means of a tracheal cannula, BAL was performed with 1 ml PBS containing a protease inhibitor cocktail (Roche, Basel, Germany). An automated CasyTT cell counter (Schaerfe Systems, Reutlingen, Germany) was used to determine total leukocyte cell counts and cell-free supernatants were stored at −20°C for cytokine quantification. For differential cell counts, cytospins were prepared, fixed, and stained with Diff-Quick (Merz & Dade AG, Dudingen, Switzerland). Standard microscopical and morphological criteria were used to identify cell types (Olympus BH2, Hamburg, Germany; Assistant 345 counter AC-8, Hecht-Assistent, Sondheim, Germany).

### Lung Histology and Immunofluorescence

Directly after performing the BAL, lungs were fixed with 10% formalin *via* the trachea, removed, and stored. Fixed lung tissue was then embedded in 2% agarose surrounded by paraffin and 3 µm thick sections were stained with periodic acid/Schiff (PAS). PAS-stained sections were microscopically assessed and random images were collected using the 10× magnification (20). Numbers of PAS+ mucus-producing goblet cells in the bronchial epithelium were recorded using five random distant airway pictures per mouse lung and were quantified per millimeter of basement membrane. For immunofluorescence analyses, tissue sections were deparaffinized and blocked with PBS containing 3% BSA and 0.3% Triton-X-100. Sections were incubated overnight at 4°C with primary antibodies against Grx2 (Abcam, UK) and F4/80 (BioLegend, San Diego, CA, USA). Sections were washed and incubated for 1 h at room temperature with secondary antibodies conjugated to Cy3 and Cy5 (Merck-Millipore, USA), respectively. Sections were washed and nuclei were stained with Hoechst (Sigma-Aldrich, Germany). Following another round of washing, sections were mounted using Immu-Mount (Thermo Scientific, USA). Tissue sections were analyzed using the Olympus BX51 Fluorescence Microscope.

### Airway Resistance (AR)

Lung function analysis was performed using non-invasive head-out body plethysmography 24 h after the last aerosol challenge. The mid-expiratory airflow of bronchial responsiveness to β-methacholine (MCh_50_) was measured as described previously (21). Briefly, the system consists of a glass made head-out body plethysmograph attached to an aerosol exposure chamber (Forschungsstaetten, Medical School Hannover, Germany). Mice are positioned in the head-out body plethysmograph while the head of the animal protruded through a neck collar (9 mm inner diameter, dental latex dam; Roeko, Langenau, Germany) into the aerosol exposure chamber, which was ventilated by continuous airflow of 200 ml/min. For airflow measurement, a calibrated pneumotachograph (PTM 378/1.2; Hugo Sachs Elektronic, March-Hugstetten, Germany) and a differential pressure transducer (8T-2; Gaeltec, Dunvegan, Scotland) coupled to an amplifier (HSE-IA; Hugo Sachs Elektronic) were attached to the top port of each plethysmograph. For each animal the amplified analog signal from the pressure transducer was digitized *via* an A/D converter (DT301 PCI; Data Translation, Marlboro, MA, USA) at a sampling rate of 2,000/s. Notocord hem 3.5 (Notocord, Paris, France) was used for data calculation. During continuous assessment of EF_50_, mice were exposed to increasing MCh aerosol concentrations (12.5, 25, 50, 75, 100, or 125 mg/ml), and airway reactivity was expressed as the concentration of methacholine that caused a 50% reduction in baseline midexpiratory airflow (MCH_50_ mg/ml).

### SDS Page and Western Blot

Determination of total protein was done using Bradford-reagent (Bio-Rad Laboratories, Hercules, CA, USA). SDS-PAGE and Western blot for Grx2 in patient samples were run using the mini-Protein TGX stain-free gels (4–20%, Bio-Rad), and PVDF membranes (Macherey & Nagel) according to manufacturer’s instructions. Following semi-dry blotting, membranes were blocked with 5% milk powder and 1% BSA in TBS, supplemented with 0.05% Tween 20, for 1 h at room temperature and were incubated with primary antibody overnight at 4°C. Following extensive washing with TBST, membranes were incubated with horseradish peroxidase conjugated secondary antibodies (Bio-Rad). Western blots were finally developed by the enhanced chemiluminescence technique. For band quantification ImageJ 1.5.2a (NIH, USA) was used and band intensity calculated by Graph Pad Prism 5 (La Jolla, CA, USA).

### Cytometric Bead Array

To quantify cytokines in the BAL, 50 μl of cell free undiluted supernatant from each sample was analyzed using mouse cytometric bead array flex sets (M60-009RDPD, Bio-Rad) according to the manufacturer’s instructions. Individual standards were reconstituted and serially diluted in a 96-well plate and all samples assessed by means of specific antibody-coated 7.5-mm capture beads and phycoerythrin-conjugated detection antibodies. Cytokine measurements were performed using the BioPlex-200 (Bio-Rad). Respective standard curves and cytokine concentrations were calculated with the BioPlex Manager Software 6.1 (Bio-Rad).

### RAW264.7 Cell Culture and Determination of Nitric Oxide (NO)

The macrophage line RAW264.7 cells were maintained in RPMI 1640 Medium (Gibco, Germany) containing 10% heat-inactivated endotoxin-low fetal bovine serum (Biowest, Nuaillé, France), 2 mM L-Glutamine, 100 U/ml penicillin, and 100 µg/ml streptomycin at 37°C and 5% CO_2_ in a humidified atmosphere. NO-analysis, the cells were cultured on phenol red-free Optimem medium (Gibco, Germany). NO-production was estimated by measuring the amount of nitrite, a stable metabolite of NO, using the Griess reagent kit (Invitrogen, Carlsbad, CA, USA), following stimulation with cytokines [TNFα (1 ng/ml), IL-1β (1 ng/ml), IFNγ (1,000 U/ml)] and respective recombinant thioredoxin family proteins (10 µg/ml) in 96-well plates for 24 h. Fifty microliters of supernatant were incubated with 10 µM NADPH (Sigma) and 0,2 U/ml nitrate reductase (Sigma) for 45 min at 37°C. Fifty microliters of Griess reagent were added and nitrite levels were determined at 562 nm using the Genios Pro plate reader (Tecan). Sodium nitrite was used to prepare the standard curve for quantification. The NO data were correlated to the total cell number, analyzed by CellTiter Blue assay (Promega) that was performed according to the manufacturer instructions.

### Patient Samples

Patients with clinically confirmed bronchial asthma and healthy volunteers were recruited by the Clinic for Internal Medicine—Department for Pneumology, University Medical Center Marburg. Human subjects participating in this study signed informed consent to participate in this research. Asthma was diagnosed by symptoms in conjunction with airway hyper responsiveness (metacholine challenge). Blood was drawn from subjects into a 9 ml Serum Z Monovette (Sarstedt). Upon centrifugation at 800 × g for 30 min at ambient temperature, the serum was aspirated and stored at −80°C until analysis.

### Statistical Analysis

Graphing and statistical analysis of normally distributed data was performed using Graph Pad Prism 5. Data are expressed as mean ± SEM or as mean and percentiles. Statistical significance was determined using the one-way ANOVA with Tukey’s Multiple Comparison Test (for multiple group comparison) or the Student’s unpaired t-test for two group comparison. Statistical significance was referred to as *p < 0.05, **p < 0.01, ***p < 0.001.

## Results

### Members of the Thioredoxin Family Are Altered in Cellular Distribution and Total Concentration Upon Airway Inflammation

**Figure 1:**
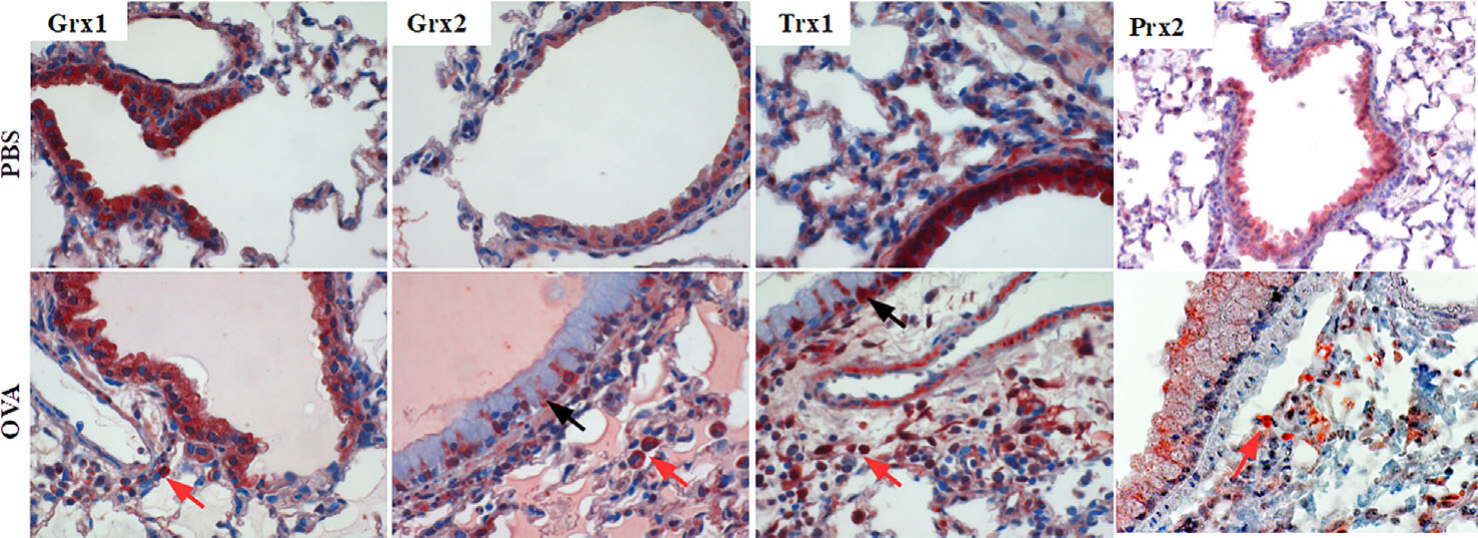
Expression pattern of Thioredoxin 1, Glutaredoxins 1 and 2, and Peroxiredoxin 2 in mouse lungs. Control lungs (PBS) and inflamed ovalbumin-exposed lungs (OVA) were analyzed by immunohistochemistry. The protein distribution patterns of Trx1, Grx1, Grx2, and Prx2 are shown on representative lung tissue cross sections of small airways (magnification 400×; black arrows, epithelium; red arrows, infiltrating cells).

Airway inflammation in mice was induced by intra-peritoneal sensitization (days 0, 7, 14) and aerosol inhalation (challenge-phase, days 25–27) of OVA as previously described (**Supplementary Figure 1**, groups PBS, OVA) (22). Sham-treated PBS mice displayed an evenly but specific staining pattern for Grx1 and 2, Trx1, and Prx2 in epithelial cells (**Figure 1**, upper lane). Upon OVA-immunization (**Figure 1**, lower lane) infiltrating cells located closely to the small airways are strongly positive for Grx1 and 2, Trx1, and Prx2. While Grx1 expression remained evenly distributed in the epithelial cells comparable to PBS control, Trx1 and Grx2 displayed similar redistribution pattern upon OVA immunization and were strongly increased in basal and mucus secreting club cells. Prx2 displayed a ubiquitous dotted and decreased staining pattern compared to the PBS control.

### Administration of Recombinant Trx1 and Grx2 Prevented Airway Inflammation and Goblet Cell Induction

**Figure 2:**
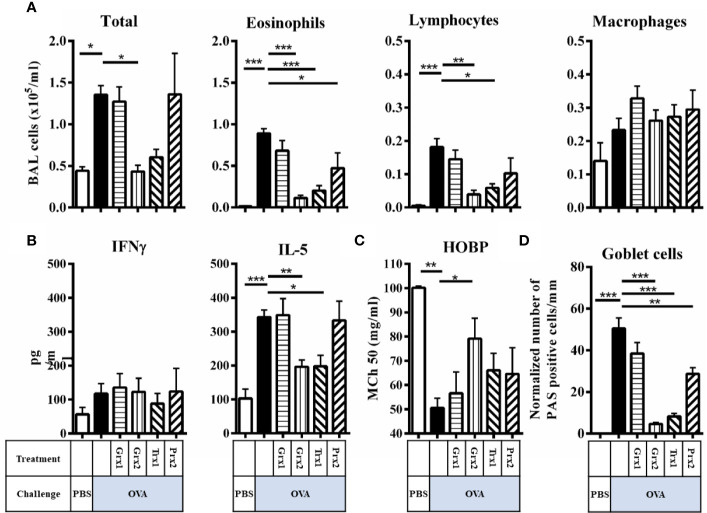
Trx1 and Grx2 protect against development of airway inflammation. **(A)** Total and differential cell quantification in the BAL of Balb/c mice subjected to ovalbumine-induced airway inflammation. **(B)** Concentrations of the cytokines IFNγ and IL-5 were measured in the BAL fluid. **(C)** Assessment of airway hyper responsiveness was measured by head-out body plethysmography (HOBP). Changes in respiratory mechanics were analyzed 24 h following three challenges of aerosolized OVA. **(D)** Quantification of mucus-producing goblet cells on lung histology sections. Shown are means ± SEM. n = 8–16 per group. Significant comparisons between PBS control animals to the OVA group and between OVA to the protein-treated OVA group are indicated. *p < 0.05, **p < 0.01, ***p < 0.001.

Next, we analyzed purified Trx1, Grx1, Grx2, and Prx2 towards their *in vivo* anti-inflammatory potential. To avoid unspecific bystander effects by pyrogenic contaminations, all proteins were subjected to endotoxin removal procedure (see **Supplementary Figure 2**). As depicted in **Supplementary Figure 1**, respective proteins were administered intraperitoneal on 5 consecutive days (days 23–27) before and during the challenge phase. To characterize the impact of the redoxin treatment we analyzed lung morphology and potential tissue remodeling by PAS-staining paralleled by characterization of infiltrating immune cells, cytokine levels, and *in vivo* lung function analysis (**Figure 2**). As expected, OVA sensitization/challenge led to a profound induction of goblet cell hyperplasia and infiltration of inflammatory cells (**Figure 2**, OVA black bars) as compared to PBS control (**Figure 2**, PBS white bars). Grx1 or Prx2 treatment did not improve visual OVA-induced histological parameters such as eosinophilia or goblet cell hyperplasia (**Figure 2**, OVA/Grx1, OVA/Prx2) similar to sham-treated OVA-sensitized/challenged animals. In contrast, Grx2 or Trx1 treatment strongly decreased cell infiltration and mucus formation on the epithelia surface similar to the PBS controls (**Figure 2**, OVA/Grx2, OVA/Trx1).

Next, we addressed whether respective redoxins are able to abolish or attenuate a type-2 driven airway inflammation in an experimental setup. To do so, we further analyzed infiltrating cells in the BAL, cytokines, and airway hyper responsiveness. Compared to the induction of inflammatory cells in the BAL by OVA, Grx2 and Trx1 showed similar potency to suppress inflammatory parameters such as total BAL cells (**Figure 2A**) (PBS 0.44·10^5^/ml, OVA 1.36·10^5^/ml, Grx2/OVA 0.43·10^5^/ml, Trx1 0.6·10^5^/ml), suppression of eosinophilia (PBS 0·10^5^/ml, OVA 0.89·10^5^/ml, Grx2/OVA 0.11·10^5^/ml, Trx1 0.2·10^5^/ml), and infiltration of lymphocytes (PBS 0·10^5^/ml, OVA 0.18·10^5^/ml, Grx2/OVA 0.04·10^5^/ml, Trx1 0.06·10^5^/ml). Grx1 or Prx2 did not suppress the inflammatory response such as infiltration of inflammatory cells in the BAL, and IL-5 production compared to OVA. Numbers of macrophages were not changed significantly by any protein analyzed in this study. While Th1 related cytokine IFNγ was not affected by any protein analyzed, IL-5 was significantly reduced by both Trx1 and Grx2 (PBS 100 pg/ml, OVA 342 pg/ml, Grx2/OVA 196 pg/ml, Trx1 197 pg/ml) (**Figure 2B**). Functionally, airway hyper responsiveness was significantly improved compared to the sham-treated OVA group (MCh_50_ 50.5 mg/ml) only by Grx2 (MCh_50_ 79.1 mg/ml) (**Figure 2C**), which correlates with decreased formation of PAS+ mucus-secreting club cells by application of Grx2 (4.6/mm), but also by Trx1 (8.3/mm) and Prx2 (28.9/mm) compared to OVA (50.4/mm) (**Figure 2D**).

### The Protective Effect of Grx2 Depends on Its Redox Activity

**Figure 3:**
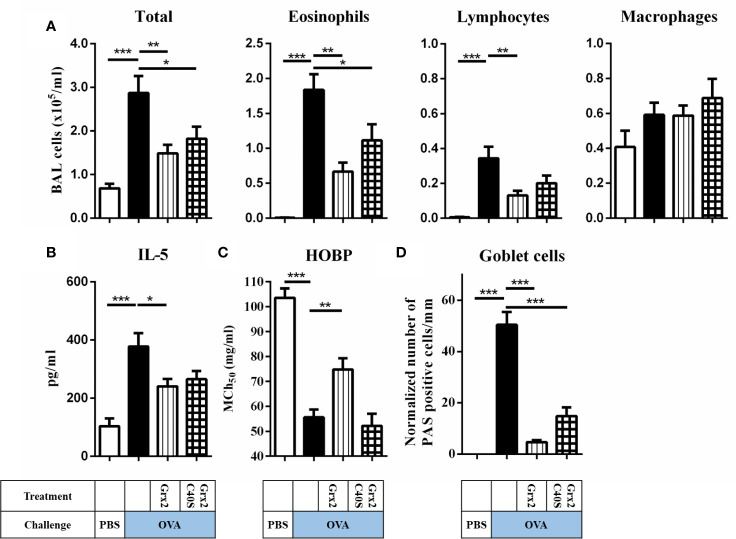
Administration of enzymatically active Grx2 catalyzing the dithiol mechanism permits the protective effect on OVA-induced allergic airway inflammation. **(A)** Total and differential cell quantification in the BAL. **(B)** IL-5 cytokine concentration in the BAL fluid. **(C)** Assessment of airway hyper responsiveness was measured by head-out body plethysmography (HOBP). Shown are means ± SEM, n = 12–16 per group. Significant differences between PBS to OVA group and OVA to protein-treated OVA group are indicated. **(D)** Representative lung tissue cross section from mice following OVA sensitization/challenge and Grx2C40S treatment stained with PAS. *p < 0.05, **p < 0.01, ***p < 0.001.

Next, we aimed at identifying the enzymatic mechanism enabling Grx2 to exert its protective functions. We expressed and purified the C40S mutant that lacks the C-terminal active site Cys residue and thus is not able to catalyze the dithiol but still the monothiol reaction mechanism (23). In contrast to Grx2/OVA treated mice lungs (see **Figure 2**), treatment using the Grx2C40S mutant resulted in formation of mucus producing goblet cell staining and inflammatory cells comparable to Grx2 WT (**Figure 3D**). Analyzing the total BAL and eosinophil cells in OVA/Grx2C40S-treated animals, we detected that even though protective effects are still significant compared to the sham-treated OVA group (p = 0.034), they are less pronounced compared to the wild type Grx2 treatment (p = 0.0021) (**Figure 3A**). The levels of pro-inflammatory IL-5 cytokine (Grx2/OVA: 240.7 pg/ml compared to OVA: 377 pg/ml) as well as airway function (MCh_50_ OVA: 55.6 mg/ml, MCh_50_ Grx2/OVA: 74.8 mg/ml) are only significantly reduced by wild type Grx2, but not by Grx2C40S (**Figures 3B, C**). To summarize, direct comparison between Grx2 WT and Grx2 C40S treatment resulted in significant difference in eosinophilia (p = 0.03) and HOPB (p = 0.0019).

### Administration of Grx2 Affected Macrophage Activation

**Figure 4:**
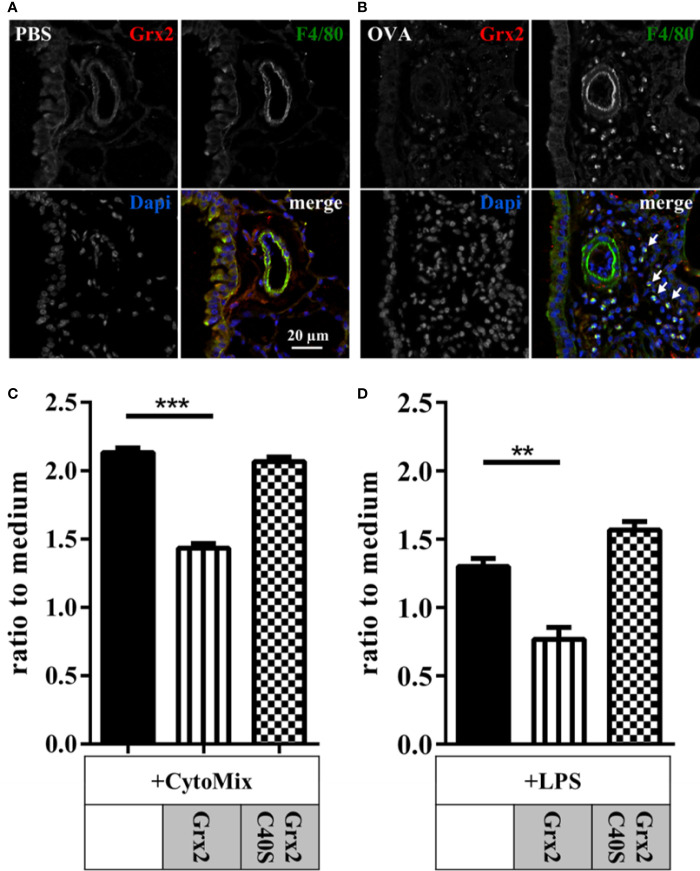
Macrophage driven inflammation is regulated by enzymatically active dithiol Grx2. Control lungs (PBS) **(A)** and inflamed ovalbumin-exposed lungs (OVA) **(B)** were stained for Grx2 and the macrophage-marker F4/80 using immunofluorescence. **(C, D)** Macrophage activation in murine RAW264.7 macrophages by cytokine mixture (Cytomix: IFNγ, IL-1β, and TNFα) or lipopolysaccharide (LPS) with or without additional treatment with either Grx2 or the respective Grx2C40S mutant. NO release, as marker for cell activation, was quantified by determining extracellular nitrate levels by the Griess reagent **(C)**. n = 3 experiments each performed in triplicates. **p < 0.01, ***p < 0.001.

Our histology data showed that infiltrating immune cells were positive for Grx2 in OVA-treated animals. Treatment with both wild type and the Grx2 mutant that cannot catalyze the dithiol reaction mechanism reduced the numbers of all analyzed immune cells in bronchoalveolar lavage, apart from macrophages (**Figures 2A** and **3A**). Since macrophages are known to be regulated by extracellular redoxins, we were interested if Grx2 affects macrophage activation. First, we verified if infiltrating macrophages were positive for Grx2 by immunofluorescence using specific antibodies against Grx2 and the macrophage marker F4/80 (**Figures 4A, B**). F4/80 positive macrophages could barely be detected in PBS-treated control lungs. However, we could demonstrate the presence of infiltrating macrophages in the lung tissue of OVA-exposed animals. Noteworthy, these infiltrating cells were positively stained for Grx2. Next, we investigated whether the administration of Grx2 directly affects the activation of macrophages. Cells of the immortalized murine macrophage cell line RAW264.7 were treated with cytokines that are known to act in the pathology of asthma (CytoMix), i.e. IFNγ, IL-1β, and TNFα in the presence or absence of recombinant Grx2 WT or Grx2 C40S. In addition, we stimulated macrophages with Lipopolysaccharide (LPS) in the presence or absence of recombinant Grx2 WT or Grx2 C40S. Following a 24 h treatment, NO release was measured using the Griess reagent. Since redoxins are also known to regulate cell proliferation, we performed the CellTiter Blue assay and adjusted NO release to similar cell numbers. Cytokine or LPS treatment induced the production and release of NO (**Figures 4C, D**). Interestingly, only the wild type Grx2 was able to reduce the NO levels to control levels.

### Serum Levels of Grx2 Are Reduced in Allergic Asthmatics

**Figure 5:**
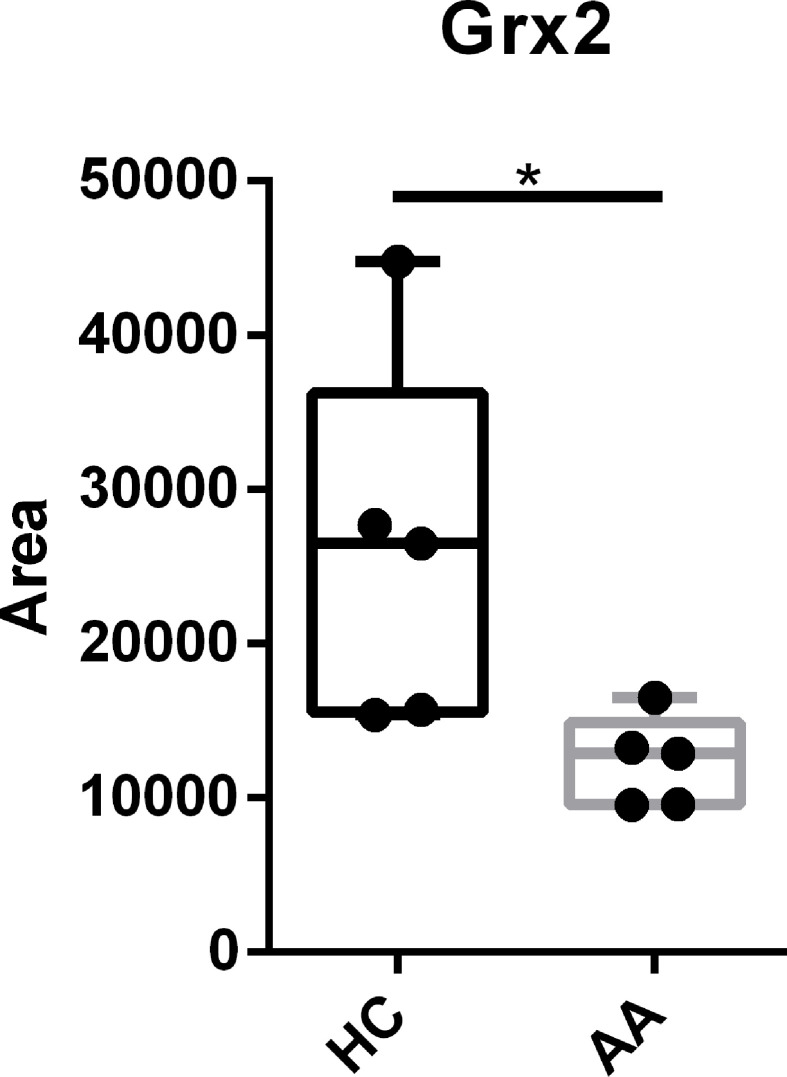
The level of Grx2 is significantly reduced in serum of allergic asthmatics compared to healthy controls. Sera from confirmed asthmatics (AA) and healthy controls (HC) where subjected to Grx2 western blot quantification. n = 5, *p < 0.05.

Since administration of Grx2 showed a protective effect in the manifestation of airway inflammation, we analyzed the levels of Grx2 in serum samples of healthy human volunteers and patients with clinically confirmed bronchial asthma. Serum samples were analyzed by SDS Page and Western Blotting using specific antibodies against Grx2. Band intensities were analyzed using Image J. Band quantification in **Figure 5** shows a significant reduction of Grx2 in the serum of allergic asthmatics compared to healthy control subjects, indicating a possible positive impact of external applied Grx2, as presented here in the mouse model.

## Discussion

Allergic asthma is a mainly type-2 helper T-cell (Th2)-driven inflammatory disease that is characterized by activation and infiltration of eosinophils, lymphocytes, macrophages, and mast cells into the lung followed by the release of specific reactive oxygen and nitrogen species which lead to protein oxidation (2–4). Oxidoreductases of the Trx family are known to regulate cellular functions *via* rapid, specific, and reversible modifications of cysteinyl residues. Their protein levels and enzymatic activities have been shown to be altered in different pathologies, including cancer, neurodegenerative as well as ischemia-induced disorders and also in pathologies related to inflammation (5).

In this study, we analyzed the distribution and therapeutical impact of four cytosolic oxidoreductases, i.e. Trx1, Grx1, Grx2, and Prx2 that all function in redox-mediated signal transduction and inflammation. Interestingly, these proteins have different subsets of substrates and catalyze different reactions (5). Trx1 is the founding member of the protein family. It was both enriched in non-ciliated club cells and infiltrating inflammatory cells in the OVA-animals. Moreover, our study confirms the therapeutical potential of administration of Trx1. Application of Trx1 efficiently reduced cell infiltration into the lung. Further, while Th1 cytokines such as IFNγ were not affected, IL-5 was significantly reduced in the BAL. Ultimately, lung function using the head-out body plethysmography was significantly improved by Trx1 (p = 0.05). Ichiki and coworkers showed that administration of Trx1 had beneficial effects in animal studies using the OVA-based mouse model of asthma-like allergic airway inflammation. Trx1 administration resulted in reduced airway hyper responsiveness and inflammation (24). Suppression of the CC chemokine-induced chemotaxis of eosinophils and increased expression of Th1 cytokines like IFNγ—which counteracts Th2 activities—seem to be key targets (24). In our study, Trx1 was administered during the sensitization phase. Equally, Ichiki et al. administered similar Trx1 concentrations parallel to the sensitization. Similar data were obtained in transgenic mice over-expressing Trx1 (25). Moreover, administration of Trx1 was also shown to protect mice from bleomycine- or cytokine-induced acute interstitial pneumonia and bleomycin-induced lung injury (26). These findings suggest that Trx1 can function in suppressing immune cell infiltration and epithelial damage. However, mechanistic studies to understand the underlying relevant molecular pathways are to date missing. Trx1 has many substrates including cytosolic 2-Cys Prxs such as Prx2. We could show that Prx2 was elevated in OVA-induced airway inflammation and it was proposed to play a protective role due to its function as peroxidase. Administration of Prx2 does not improve all analyzed individual inflammatory parameters. However, we could detect a positive impact of Prx2 treatment on the overall lung function. For Prx1 a suppression of IL-2 and a direct regulation of the Th1/Th2 balance has been suggested, Prx2, however, appears to have an indirect impact *via* the regulation of the B-cell activation factor (12). Prx1 and 2 have been shown to bind and activate TLR4 receptor-mediated and signaling and have been described as DAMPs and PAMPs in the extracellular space (27). We believe that Prx2 mainly functions in redox-mediated signal transduction under inflammatory conditions rather than as peroxidase. Reduction of peroxides would imply the abolishment of pathological conditions, whereas administration or increase of peroxides would enhance these conditions. Interestingly, administration of superoxide dismutase (SOD) that leads to the formation of hydrogen peroxide, showed beneficial effects in a guinea pig model of OVA-induced airway inflammation (28). Moreover, transgenic mice ubiquitously overexpressing catalase showed a similar degree of airway and tissue inflammation when subjected to OVA, and developed an increased airway hyper responsiveness to methacholine. The authors suggested that hydrogen peroxide functions in the suppression of mucus production and airway hyper responsiveness, rather than being the cause (29). Knock-out of eosinophil peroxidase to assess the role(s) of this abundant secondary granule protein in an OVA-challenge model had no effects on OVA-induced pathologies in the mouse lung (30). Because Prxs are peroxide sensors and transducers of oxidation, it is tempting to speculate that they regulate crucial pathways. Prxs are regenerated in NADPH-dependent reactions catalyzed by Trx or Grx.

We also tested the distribution of Grx1 and Grx2. Distribution of Grx1 is, similar to Prx2, not affected during airway inflammation and has no effect on the parameters analyzed compared to OVA mice. Grx1 was increased in sputum of patients with COPD (10) and asthmatics, as well as in the OVA-based asthma mouse model (31). Grx1 was previously shown to be up-regulated on mRNA and protein level in the OVA-induced airway inflammation mouse model (29). In primary tracheal cells, Grx1 expression was altered by administration of different cytokines and directly correlated with levels of protein glutathionylation. S-glutathionylation and extracellular GSH showed only a short-term increase at 6 h. Analyzing temporal relationships, the authors state that the induction of Grx1 leads to de-glutathionylation of proteins including e.g. the p50 and p65 subunits of NF-kB and IKK and thereby an increase in pro-inflammatory cytokines (32). More recently, Grx1 levels were shown to be increased particularly in the lung epithelium, alveolar macrophages and in the bronchoalveolar lavage (BAL) of OVA-sensitized/challenged mice between 6 h and 8 days following the last OVA-challenge. Hoffmann *et al.* have shown that genetic ablation of *GLRX1* attenuated airway inflammation in the house dust mite mouse model. Grx1 deficient mice showed a different pattern of the infiltrating immune cells as well as the released cytokines. They showed decreased eosinophil infiltration and expression, release of NF-kB regulated pro-inflammatory cytokines, and enhanced mucus metaplasia and airway hyper responsiveness. Interestingly, these mice displayed an increase in protein-glutathionylation (33). In line with results of these studies, our study clearly shows that treatment with recombinant Grx1 did not improve any of the analyzed parameters (such as eosinophilia, IL-5 release, or lung function). In a mouse model of lung fibrosis, characterized with low Grx1 activity and increased glutathionylation, administration of Grx1 to the lung was shown to exert beneficial effects (34). Glutathionylation is an oxidative modification that has been described to protect cysteines from irreversible oxidation during conditions of redox dysbalance. Moreover, glutathionylation of specific cysteinyl residues can e.g. affect nuclear translocation, protein release, and DNA binding of the transcription factor NF-kB and is regulated by Grx1 (35–37). Intravenous injection of Grx1 was able to suppress inflammation-induced neutrophil mobilization from the bone marrow *via* a mechanism that involves de-glutathionylation of integrin alpha 4 (38).

The second dithiol Grx is Grx2. Similar to Grx1, Grx2 also catalyzes disulfide exchange reactions and (de-)glutathionylation of target proteins. Here, we showed that Grx2 was also enriched in non-ciliated club cells and inflammatory cells, similar to Trx1; highlighting the importance of redox-control during destructive inflammatory airway response such as asthma (39). In contrast to Grx1, treatment with Grx2 was also able to suppress inflammatory parameters. It led to a ubiquitous reduction of inflammatory markers such as influx of eosinophils, corresponding Th2 cytokine IL-5, while Th1 cytokines remained unaffected. Ultimately, lung function using the head-out body plethysmography was also significantly improved by Grx2 (p < 0.05).

Since Grx2 was expressed in infiltrating macrophages we used the RAW264.7 cell line to further study the impact of Grx2 on macrophage function. Cytokine treatment induced the production and release of NO in these cells which was attenuated in the presence of Grx2. This function was based on the Cys-X-X-Cys active site motif, since the monothiol mutant did not inhibit NO release. NO may have been transformed into dinitrosyl-diglutathionyl-iron-complexes, a mechanism that was recently described for activated microglia (40). So far, in both mice and human subjects with allergic airway inflammation, changes of the Grx2 levels before and after sensitization were not reported.

Here, we have also included the analysis of serum samples of healthy human volunteers and patients with clinically confirmed bronchial asthma. The Grx2 levels of patients were significantly reduced. Asthma constitutes a worldwide health issue and current medications are limited. It is of clinical importance to develop new therapeutic intervention strategies and tools. In a recent review article, the authors suggest that a therapy that targets oxidizing molecules in combination with inflammatory mediators and transcription factors could have clinical significance (41). Our data clearly demonstrate a potential clinical value for Trx1, as proposed before, and Grx2 in asthma. Thus, more studies are needed to analyze if these oxidoreductases could also be used as therapies for manifested airway inflammation.

## Data Availability Statement

All datasets presented in this study are included in the article/**Supplementary Material**.

## Ethics Statement

The animal study was reviewed and approved by Regierungspräsidium Giessen, Hessen, Germany. Human BALF was obtained following the ATS consensus procedure in accordance with local ethics regulations (87/12).

## Author Contributions

EH, HG, CL, and CHu participated in the conception of the study. EH, CHe, CB, and CHu performed the experiments. CHu performed the statistical analysis. EH, CB, and CHu wrote the manuscript. BW provided human serum samples. All authors contributed to the article and approved the submitted version.

## Funding

This study was supported by P. E. Kempkes to Chu (06/2010, Marburg, Germany), the von Behring Röntgen Foundation (to CL), and the German Research Council [DFG; SPP1710 to EH (HA 8334/2-2), CB (BE 3259/5-2), and CL (SFB593-N01, GRK1947-A1, LI 984/3-1, LI 984/3-2, LI 984/4-1)].

## Conflict of Interest

The authors declare that the research was conducted in the absence of any commercial or financial relationships that could be construed as a potential conflict of interest.

## Abbreviations

Grx, glutaredoxin; OVA, ovalbumin; Prx, peroxiredoxin; Trx, thioredoxin; GSH, glutathione; BAL, bronchoalveolar lavage; Cys, cysteine; H_2_O_2_, hydrogen peroxide; COPD, chronic obstructive pulmonary disease; NO, nitric oxide; GSSG, glutathione disulfide; PAS, periodic acid-Schiff; HOBP, head-out body plethysmography.
