# Supplementary material for: Glutaredoxin 2 Reduces Asthma-Like Acute Airway Inflammation in Mice
Source: Front Immunol. 2020 Nov 3;11:561724. doi: 10.3389/fimmu.2020.561724 (PMC7670054; doi:10.3389/fimmu.2020.561724)
Supplement: Supplementary file 1 [file DataSheet_1.pdf]

Glutaredoxin 2 reduces asthma-like acute airway inflammation in mice

-Supplementary-

Hanschmann E.M.[1], Berndt C.[1], Hecker C.[1], Garn H.[2], Bertrams W.[3], Lillig, C.H.[4] and Hudemann C.* [5,6]

[1] Department of Neurology, Medical Faculty, Heinrich-Heine University Düsseldorf, Düsseldorf, Germany

[2] Biochemical Pharmacological Center (BPC) - Translational Inflammation Research Division, Philips Universität Marburg, Member of the German Center for Lung Research (DZL) and the Universities of Giessen and Marburg Lung Center (UGMLC), Marburg, Germany

[3] Institute for Lung Research, Universities of Giessen and Marburg Lung Center, Philipps-University Marburg, Member of the German Center for Lung Research (DZL), Marburg, Germany

[4] Institute for Medical Biochemistry and Molecular Biology, University of Greifswald, Greifswald, Germany

[5] Department of Dermatology and Allergology, Philipps Universität Marburg, Marburg, Germany

[6] Institute of Laboratory Medicine and Pathobiochemistry, Molecular Diagnostics, Philipps University Marburg, Marburg, Germany

*** Correspondence**

Christoph Hudemann, PhD

e-mail: christoph.hudemann@staff.uni-marburg.de

phone: (+49) 6421 58 64823

Keywords: airway inflammation, asthma, redoxins, oxidative dysbalance

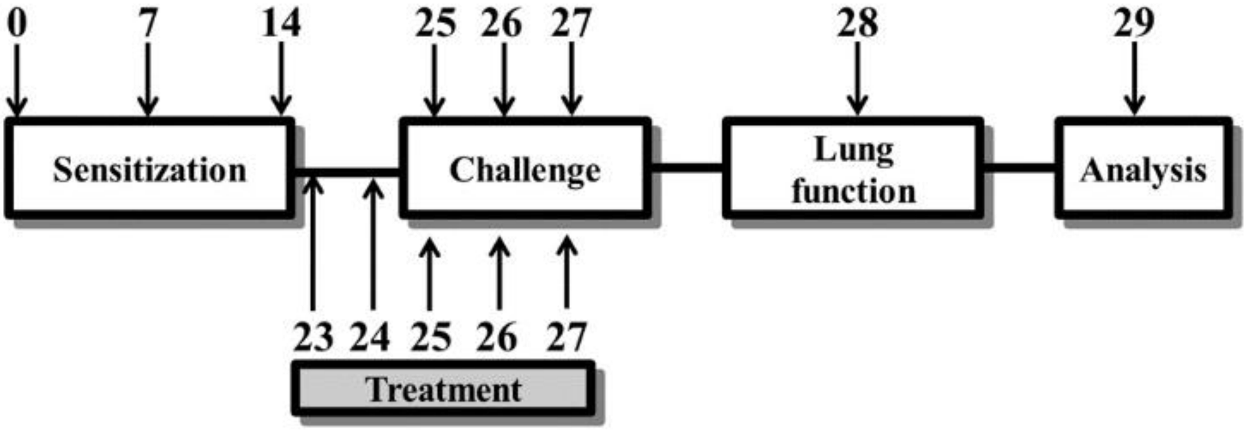

Group	Protein	Treatment (i.p.)	Sensitization (i.p.) / Challenge (aerosol)
PBS	-	-	PBS
OVA	-	-	OVA
Grx1	Grx1		
Grx2	Grx2		
Grx2 C40S	Grx2 C40S	40µg / day 100µl	OVA
Trx1	Trx1		
Prx2	Prx2		

Supplementary Figure 1: Scheme of the experimental treatment protocol for the induction of allergic airway inflammation and treatment. Female Balb/c mice were treated as indicated. Control mice received PBS i.p. as sham-sensitization and challenge by aerosol. All other mice were exposed to ovalbumin (i.p. and aerosol). Recombinant proteins were administered (i.p.) before and in parallel to the challenge phase as indicated. The analyses were performed at days 28 (*in vivo* lung function analysis) and 29. Airway inflammation was confirmed by increased infiltration of inflammatory cells, histological changes and decreased lung function. (n=10-16 in 2 experimental series, with 5-8 animals per group)

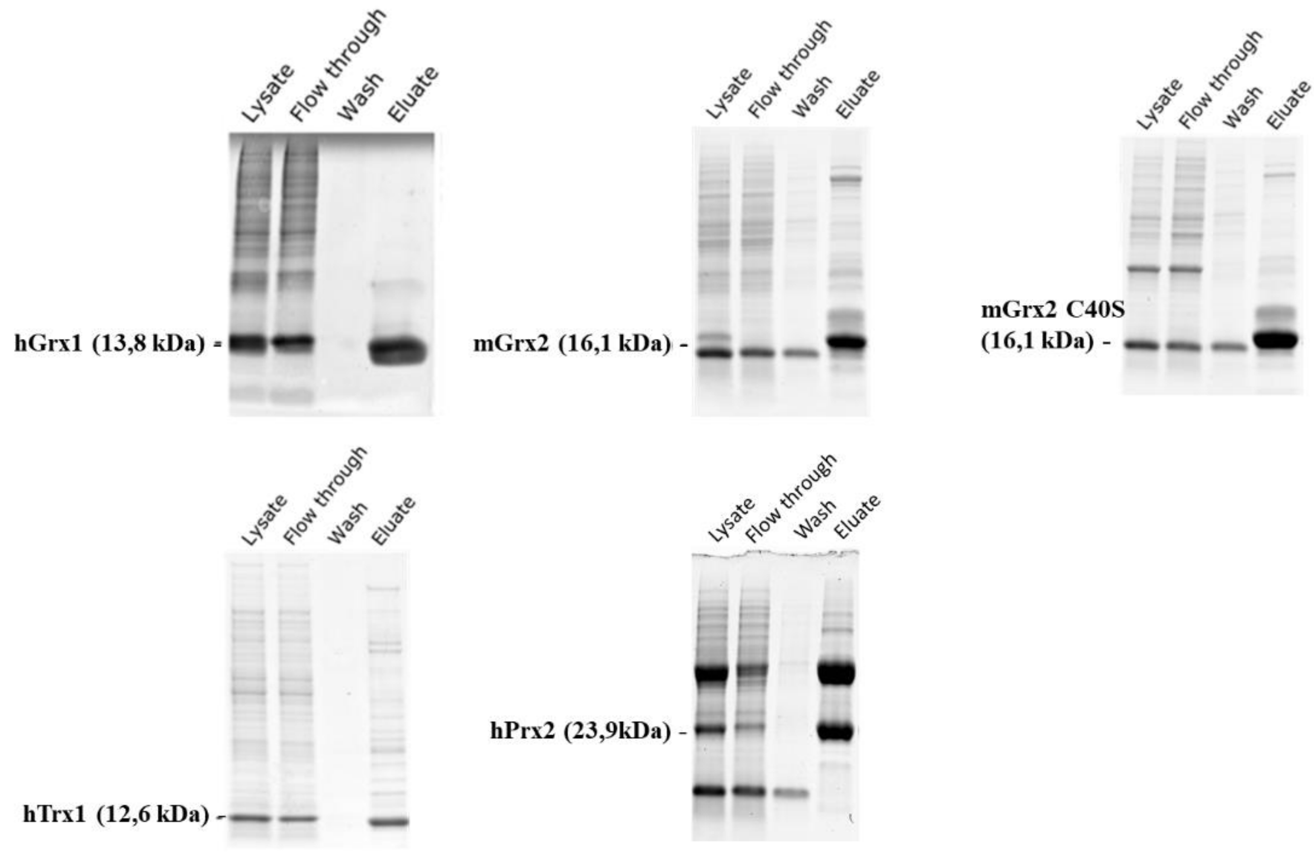

Supplementary Figure 2: Affinity purification of applied proteins. Recombinant proteins were expressed as HisTag-fusion proteins in *E. coli*. Protein purification was performed using immobilized metal affinity chromatography. Proteins of the lysate, flow through, wash fraction and elution were separated by SDS-Page and stained with coomassie to verify the purifications of Thioredoxin 1 (Trx1), Peroxiredoxin 2 (Prx2), Glutaredoxin 1 (Grx1) and 2 (Grx2) and the mutant Grx2 C40S. Sizes of the monomeric proteins are indicated.

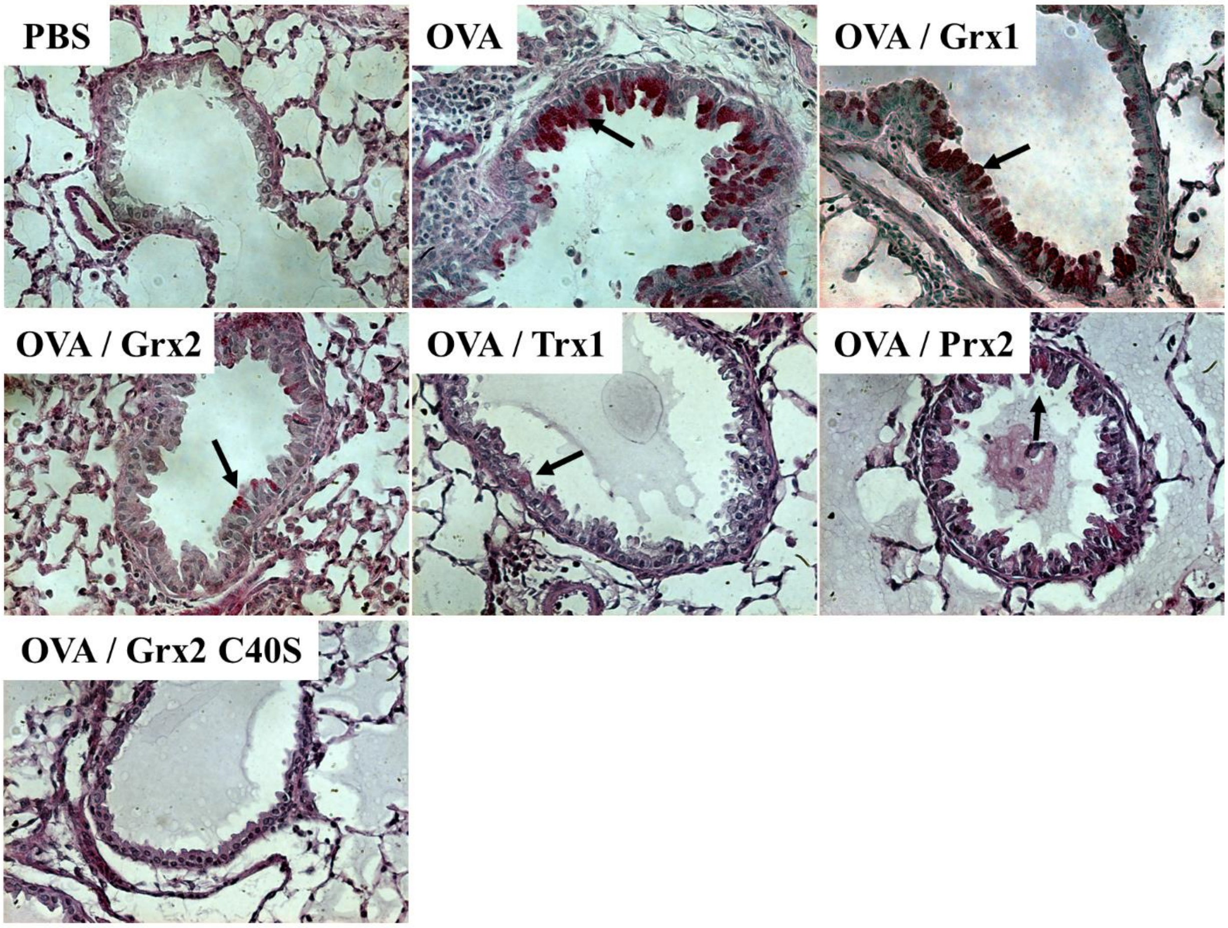

Supplementary Figure 3: Lung sections were analyzed with hematoxilin and eosin (H&E) and the periodic acid-schiff (PAS) method. Representative photomicrographs show a marked reduction of club cell formation in Trx1, Grx2 and Grx2 C40S-treated mice compared to OVA-treated animals. Original magnification x400.
